# An Undescribed Form of Stricture at the Orifice of the Male Urethra

**Published:** 1884-09

**Authors:** 


### AN UNDESCRIBED FORM OF STRICTURE AT THE ORIFICE OF THE MALE URETHRA.

BY

THE EDITOR.

IN none of the works on Surgery, general or special, which I have consulted, can I find any reference to the form of stricture which I here wish to describe. Apart from its rarity, the condition warrants a special description on account of the troubles it gives rise to and its resistance to treatment. In fact, I have met with no strictures of the urethra which gave both the patient and myself so much trouble as the two cases I have to relate. Indeed, I may say that, as compared with ordinary strictures of the urethra, the treatment of this form has been in my hands complete failure.

The first case was sent me by Mr. Skelton, of Downend. The patient was a strong, healthy labouring man, aged thirty-four, married, with children. He had never had any venereal disease, and no rashes, sores, or any irritative lesion on the glans or prepuce. During all his life, and for the first few years of his married life, he had been able to retract the foreskin completely and without difficulty. Within the past two years the foreskin had become adherent to the glans in a semi-retracted position, as the foreskin usually lay. At the same time the exposed portion of the glans became covered with a hard semi-cartilaginous tissue, which extended into the orifice of the urethra and caused narrowing of the canal. So much had this narrowing proceeded that when I saw him he was able only after violent expulsive efforts to propel the water in a tiny stream. Usually the water came only in drops, and micturition was always attended with great pain.

On examination, the whole of the mucous membrane of the glans penis almost up to the corona was replaced by a dense gristly material, so unyielding as to altogether prevent enlargement during erection. The exposed frenum was greatly enlarged and thickened. Behind the corona a probe, pushed through the adhesion between prepuce and glans, could be moved all round the penis. The meatus was contracted to a pin-point, admitting with difficulty an ordinary surgical probe, and was surrounded with the same dense, unyielding, gristly material. The contraction seemed to extend about a third of an inch down the urethra.

I divided the adhesions between glans and prepuce, finding the corona free from adhesions; slit up the orifice so as to admit a No. 10 English catheter, and tried to dissect the gristly material from the glans. But this last I could not perfectly do, as the tissue extended into the gland substance, and there was no line of demarcation between it and the erectile tissue. After a few weeks it was evident that I had effected no improvement. The surface of the glans got as hard as ever, and the induration along the margin of the prepuce reappeared. But what was most annoying was that, in spite of constant passing of bougies, the meatus speedily contracted again. The passage of a large instrument caused more pain than the patient, though he was a plucky fellow, could bear, and the result was that he was discharged with a No. 4 French rubber bougie, which he passed once or twice daily. He attended some months as an out-patient, and it was only by steady perseverance, in spite of great pain, that he has been able to keep the calibre of the urethra up to the small size it now is.

I need not say that he had all sorts of emollient applications—vaseline, glycerine, iodide of potassium ointment, and so forth—all of them useless. All the treatment I could apply did him little good; in fact, as the hardening since division of the adherent prepuce has extended backwards, it may have done harm. He passes a slightly larger stream by the help of the bougie, but the pain from the bougie is almost as great as the pain used to be from micturition through the pin-hole orifice.

My second case, a strong, healthy lad of eighteen, was also an Infirmary patient. He was admitted in May, 1883, complaining of pain and great difficulty in micturition. The urine came in a very small stream or in drops, and he took about ten minutes in emptying the bladder. This had been going on and gradually getting worse for two years. He had had no venereal disease, and up till his troubles began he had had no difficulty in fully retracting the foreskin.

His condition was almost exactly similar to that of the previous case, except that the prepuce was adherent to the glans closer to the meatus, less than half-way between it and the corona; and at one side of the frenum a deficiency in the adhesion easily admitted a probe, which could be freely moved in the cavity so left. There was the same gristly condition of the frenum, and the same extension of the thickening for a little way down the urethra.

The patient would not submit to ætherization, and so no operative procedure was possible. The parts were kept soaking in glycerine, and attempts made to dilate the orifice by bougies. This caused so much pain, however, and the results were so slow and so doubtful, that the patient left when the orifice would admit with difficulty a rubber bougie about the size of an English No. 3. I have seen or heard nothing of the lad since.

What is the nature of this condition? That the stricture of the meatus is of the same nature as the thickened and contracted mucous membranes generally is evident enough. But deeper than this into the nature of the malady it is difficult to go. At first phimosis, and the irritation consequent thereon, suggests itself. But neither patient had phimosis, and the adhesions were least where those in phimosis are usually greatest, namely, over and behind the corona glandis. Repeated attacks of Herpes Progenitalis might be offered as a cause. But both patients denied having suffered from this, or any other eruptive or ulcerative malady whatever. Both maintained that their complaints came on of their own accord, without any apparent cause.

In fact, to presuppose any inflammatory condition would by no means get rid of the difficulty. Nothing less than a severe scald would beget dense cicatricial tissue so evenly distributed over the whole surface; and no traumatic or inflammatory condition which I have ever seen has left so even a surface behind it. In one of the cases the sclerosis and urethral contraction went on steadily under a medical man's eye without the appearance of any inflammation whatever. Mechanical irritation, long continued, might doubtfully start such a condition, but even then the mischief would not go on increasing after the irritation had certainly been stopped.

I believe that, on the whole, we must regard the condition as a true cirrhosis of mucous membrane, as scleroderma is of the skin. It was clearly not a mere epidermic hypertrophy; the sclerosis deeply involved the fibrous tissues of the glans. The dense tissues were anæmic and transparent, as in scleroderma, and exhibited no line of demarcation from the underlying substance.

The local condition—the pale, glistening, hard, contracted dermis, and the history, owning no irritative or other exciting cause, tally readily enough with the disease scleroderma; but the situation, on mucous membrane, and the existence of adhesions—not very strong, certainly, but still palpable enough—between orifice of prepuce and glans, are against its being true scleroderma, though not insuperably so. If it is not a scleroderma, I can offer no further suggestion as to what it is.

Whatever be its true pathology, there can be no doubt as to the reality and urgency of its symptoms. The pain during micturition is far more severe than we find in cases of ordinary stricture, and the measure of relief which I have been able to give, after much thought and trouble, has been very small. Removal of the contracted orifice would be so much of an experiment that I should not dare to propose it. And yet, if the spontaneous cure which we occasionally see in cases of pure scleroderma does not take place here, I know of no other means of relieving these patients from their sufferings.

